# The influence of Mg/Al molar ratio on the performance of CuMgAl-x catalysts for CO_2_ hydrogenation to methanol

**DOI:** 10.3389/fchem.2024.1361930

**Published:** 2024-03-14

**Authors:** Haoran Liu, Wenbin Huang, Zhen Xu, Yijing Jia, Meng Huang, Xiaoyue Liu, Han Yang, Rongrong Li, Qiang Wei, Yasong Zhou

**Affiliations:** State Key Laboratory of Heavy Oil Processing, China University of Petroleum, Beijing, China

**Keywords:** CuMgAl-x catalysts, Mg/Al molar ratio, CO_2_ hydrogenation, methanol, Cu-MgO interaction, Cu^+^ species

## Abstract

The CuMgAl-x catalysts derived from hydrotalcite precursors with different Mg/Al molar ratios were synthesized and applied to CO_2_ hydrogenation to methanol reaction. In this study, the effects of Mg/Al molar ratio on the structure and surface properties of CuMgAl-x catalysts were investigated by XRD, N_2_ adsorption-desorption, SEM, TEM, H_2_-TPR, CO_2_-TPD, XPS, and *in situ* DRIFTS characterization methods. The results showed that an appropriate Mg/Al molar ratio can enhance the Cu-MgO interaction, increasing the basic sites and obtaining suitable acid sites. The dispersion of active Cu on the CuMgAl-x catalysts can be improved by strong Cu-MgO interaction, which enhances the adsorption capacity of CO_2_ and makes H_2_ activation easier, accelerates the conversion of intermediate species CO_3_^*^ and HCO_3_^*^to HCOO^*^, and facilitates further conversion to CH_3_O^*^ and CH_3_OH. The strong interaction between Cu and MgO was conducive to the formation of Cu^+^, which can inhibit the desorption of CO in the reverse water gas shift reaction. The CuMgAl-3 catalyst showed the highest CO_2_ Conversion rate (14.3%), methanol selectivity (94.5%), and STY of methanol (419.3 g⋅kg_cat._^−1^⋅h^−1^) at 240°C and 2.5 MPa. The results obtained in this paper can provide a new idea for the design of high-performance catalysts for CO_2_ hydrogenation to methanol.

## Graphical Abstract

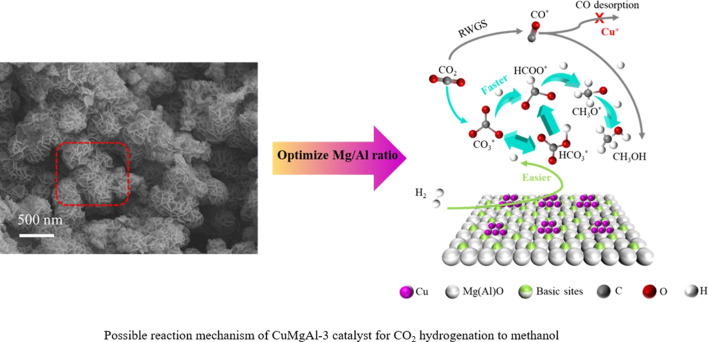

## 1 Introduction

In recent decades, excessive carbon dioxide emission has become a serious problem leading to global climate change, causing great harm to human survival (Du et al., 2016; Kumar and Kim, 2016; Dong et al., 2017). Converting CO_2_ into methanol by using green H_2_ is one of the ways to achieve CO_2_ emission reduction and effective utilization, which has attracted great attention from scholars in recent years (Lei et al., 2016; Xiao et al., 2017; Zhang et al., 2017; Chen et al., 2022). Methanol is an important raw material for many value-added chemicals and can be used as a fuel additive or clean fuel, methanol can also be converted into high-octane gasoline, aromatics, ethylene, propylene, and other petroleum chemicals (Ren et al., 2015; Fang et al., 2019a). Cu-based catalysts have been widely used in the study of CO_2_ hydrogenation to methanol. Scholars generally believe that relatively low-cost copper-based catalysts are promising candidates for carbon dioxide hydrogenation to methanol (Song et al., 2023a; Han et al., 2023; Zhang et al., 2023). However, Cu-based catalysts still face the problem of low activity and low stability (Xu et al., 2022; Chen et al., 2023). Therefore, it is of great significance to develop Cu-based catalysts with high activity and excellent stability for CO_2_ hydrogenation to methanol.

Recently, layered double hydroxides (LDHs) have attracted more and more attention due to their excellent physical and chemical properties (Karim et al., 2022; Zhang et al., 2024a). The classical formula of LDHs is ${\left[M_{1-x}^{2+}M_{x}^{3+}{\left(OH\right)}_{2}\right]}^{z+}•{\left[A_{\frac{z}{n}}^{n-}•mH_{2}O\right]}^{z-}$ , in which M^2+^and M^3+^are divalent and trivalent metal cations (Teixeira et al., 2018). Composite oxides derived from LDHs have the advantages of uniform dispersion of M^2+^and M^3+^at the atomic level, synergistic effect between elements, large specific surface area, strong basicity, and high anti-sintering stability, which are widely used in the CO_2_ hydrogenation conversion process (Fang et al., 2019b; Wierzbicki et al., 2020; Zheng et al., 2021). Gao et al. (2013, 2015, 2016) synthesized a series of CuO-ZnO-Al_2_O_3_-ZrO_2_ catalysts through hydrotalcite-like precursor, improving the catalytic performance of CO_2_ conversion and methanol selectivity, and confirmed the advantages of Cu-ZnO-Al_2_O_3_-ZrO_2_ catalyst derived from LDH precursor in CO_2_ hydrogenation. Cored J et al. (2022) prepared Cu/MgO/Al_2_O_3_ mixed oxide catalyst with CuMgAl hydrotalcite as the precursor and applied it to the hydrogenation of CO_2_ to methanol. The catalyst contains small Cu nanoparticles with a narrow distribution (2 nm). The remarkable activity of this copper-based catalyst is attributed to the lattice reorganization associated with the water-promoted “HT memory effect,” which is beneficial to the stability of Cu^+^ ions under reaction conditions.

Strong metal-support interaction (SMSI) is very important for supported catalysts. SMSI plays a crucial role in determining metal particle size, metal dispersion, electron transfer, and oxygen vacancy formation, resulting in large differences in catalyst activity, selectivity, and stability. In recent years, to investigate the SMSI, many scholars have carried out a series of studies on the identification of catalytic active sites or interfaces, the establishment of structure-performance relationships, and the exploration of reaction mechanisms (Yao et al., 2019; Dharmalingam et al., 2023; Liang et al., 2023). Xu et al. (2023) adjusted the interfacial structure of the Cu/ZrO_2_ catalyst by changing the molar ratio and precipitation sequence of Cu and Zr precursors in the oxalate precipitation method and found that the interfacial structure gradually changed from the traditional ZrO_2_-Cu interface to the Cu-ZrO_2_ inverse interface with higher catalytic performance as the Cu/Zr ratio increased. Liu et al. (2021) reported a simple strategy to fabricate ZnFe_2_O_4_ spinel-supported Cu catalysts with tunable Cu nanoparticle sizes for CO_2_ hydrogenation to methanol. The Cu-ZnO interface acted as the active site to accelerate methanol generation and the activity of each Cu-ZnO site. Chen et al. (2019) found that the Cu-LaOx interface constructed by highly dispersed Cu nanoparticles loaded on a La-modified SBA-15 carrier exhibited a high selectivity of up to 81.2% for methanol and improved stability. Cao et al. (2021) identified the MgO/Cu interface as a highly active site through density functional theory (DFT) calculations and microdynamic modeling and proposed a new lattices-oxygen reaction mechanism for methanol formation at the interface.

What’s more, the valence state of Cu in the active interface is highly sensitive to methanol synthesis (Liu et al., 2020). Although Cu usually exists as a mixed valence state of Cu^0^ and Cu^+^ in hydrogenation reactions, recent studies have also proved that Cu^+^ present at the active interface is a favorable factor for methanol synthesis (Chen et al., 2023; Zhang et al., 2023). In addition, The highly dispersed copper nanoparticles are conducive to the formation of more active interfaces, so the size and dispersion of copper nanoparticles (NPs) also have a great influence on the catalytic performance (Xu et al., 2022; Han et al., 2023). Therefore, it is of great significance to develop catalysts with highly dispersed Cu NPs and a more efficient active interface to promote CO_2_ hydrogenation to methanol.

In this study, a series of CuMgAl-x catalysts derived from hydrotalcite precursors with different Mg/Al molar ratios were prepared by the coprecipitation method and supported with the same amount of Cu. The structure and surface properties of the CuMgAl-x catalysts are determined by XRD, N_2_ adsorption-desorption, TEM, SEM, XPS, H_2_-TPR, CO_2_-TPD, and *in situ* DRIFTS characterization methods. The effect of Cu-MgO interaction on CO_2_ adsorption, activation, and further conversion during CO_2_ hydrogenation to methanol was studied in detail.

## 2 Materials and methods

### 2.1 Catalyst preparation

The CuMgAl-x catalysts with different Mg/Al molar ratios were prepared by an improved coprecipitation method as to the literature reported (Xiao et al., 2017). A certain amount of Cu(NO_3_)_2_·3H_2_O, Mg(NO_3_)_2_·6H_2_O, and Al(NO_3_)_3_·9H_2_O (Mg/Al molar ratio = 1,2,3,4,5) was dissolved in 100 mL deionized water as metal salt solution A. Solution B consists of 100 mL sodium carbonate solution (0.15 M). To obtain the LDH precursor, solution A was dropped into solution B under intense agitation at 65°C. The pH during precipitation was monitored by a pH electrode and adjusted with an alkali solution of NaOH (1.0 M), a constant value of 10.0 ± 0.5. The resulting suspension was aged at 65°C for 5 h. Then the precipitate was filtered and washed with deionized water to pH 7 and dried at 100°C for 12 h. The obtained solids were the hydrotalcite-structured CuMgAl catalysts precursors. The precursors were ground to fine powder and calcined in static air at 500°C for 5 h (ramp rate of 2°C/min) in a muffle furnace. The resulting material was extruded into a circular sheet, then crushed and sieved into particles with 20–40 mesh size. The synthesized catalysts are noted as CuMgAl-x, where x (x = 1,2,3,4,5) was the Mg/Al atomic ratio of the catalyst. The content of Cu in all CuMgAl-x catalysts was 10 wt%.

### 2.2 Catalyst characterization

Using Rigaku SmartLab SE photoelectron spectrometer with Cu Kα radiation (40 kV, 40 mA) to obtain the diffraction characteristics of the prepared material. XRD patterns were recorded for 2 h values from 5° to 90° with a scanning rate of 10°C/min.

The pore structure characteristics of the synthesized materials were obtained by N_2_ adsorption-desorption method: the synthesized samples were treated at 300°C vacua for 4 h by Beishide 3H-2000PS2 physical adsorption instrument, and then cooled to −196°C for N_2_ adsorption and desorption experiments, and the changes of N_2_ adsorption and desorption with pressure were recorded. The BET equation was used to calculate the specific surface area of the samples, and the BJH equation was used to calculate the pore size distribution and pore volume of the samples.

TEM detection was carried out on the FEI Tecnai G2 F30 transmission electron microscope equipped with X-ray energy spectroscopy (EDX) with a resolution of 0.14 nm and a voltage of 210 kV. Before detection, the catalyst was reduced by H_2_, and the sample was dissolved in anhydrous ethanol and ultrasounded for 20 min. Then the sample was dropped onto a nickel net coated with a carbon film, which was thoroughly dried for testing.

The surface morphology and structural characteristics of the catalyst were ob-served by Hitachi SU8010 scanning electron microscope (SEM) with an operating volt-age of 5 kV.

H2 temperature-programmed reduction (H_2_-TPR) was carried out on AutoChem1 Ⅱ 2,920 equipped with a thermal conductivity detector (TCD) to analyze the reduction characteristics of the synthesized samples. Typically, the samples (about 150 mg) need to be pretreated under flowing argon for 2 h at 300°C to remove physically adsorbed water. As the temperature cooled down to 50°C, the samples were heated from 50°C to 600°C at a rate of 5°C/min in 10% H_2_ flow (balanced with Ar).

The surface basic sites or acid sites of the samples was analyzed by the CO_2_ and NH_3_ temperature-programmed desorption (TPD) experiment on ChemStar TPx chemisorption analyzer. 0.1 g sample was first reduced for 2 h at 300°C in a 10 vol% H_2_/N_2_ flow. After cooling to 50°C, the sample was saturated with CO_2_ or NH_3_ (30 mL/min) for adsorption for 0.5 h and then flushed with He (40 mL/min) for 0.5 h. Thereafter, the experiment was tested with a heating rate of 5°C/min under He flows.

X-ray photoelectron spectroscopy (XPS) was measured by Kratos XSAM800 spec-trometer, and the elements contained in the samples were tested by Al Kα rays (12 kV, 15 mA, hv = 1,486.6 eV). The experimental binding energies were calibrated according to C1s (284.6 eV).

Inductively coupled plasma optical emission spectroscopy (ICP-OES) was used to determine the element content in the catalyst.

The copper dispersion (D_Cu_), the specific surface area of exposed Cu in the sample (S_Cu_), and the average Cu particle size (d_Cu_) were determined by the N_2_O-TPR method. Typically, 100 mg samples were pretreated in N_2_ flow at 300°C for 1 h, then cooled down to 50°C. Thereafter, the samples were heated to 300°C at a heating rate of 5°C/min. The samples then were reduced with 10 vol% H_2_/N_2_ (30 mL/min) at 300°C for 1 h, the cor-responding consumption of H_2_ is defined as X. Then, the reduced samples were exposed to 2 vol% N_2_O/He (30 mL/min) at 60°C for 1 h, ensuring the surface metal Cu was completely oxidized to Cu_2_O. After that, the samples were purged again with N_2_ for 30 min and cooled to room temperature. Finally, the sample was heated to 300°C in a 10 vol% H_2_/N_2_ (30 mL/min) at the same temperature ramping, where the amount of H_2_ consumption was defined as Y. The D_Cu_, S_Cu_, and d_Cu_ were calculated by the following Eqs 1–3:

$$D_{Cu}=\frac{2Y}{X}\times 100\%$$ (1)

$$S_{Cu}=\frac{2Y\times N}{1.4\times 10^{19}\times W\times X}$$ (2)

$$d_{Cu}=\frac{1.1}{D_{Cu}}$$ (3)

where N represents the Avogadro constant (6.02 × 10^23^ atom mol^−1^), and W represents the atomic mass of copper (63.546 g/mol).

*In situ* diffusion reflectance infrared Fourier transform spectroscopy (*in situ* DRIFTS) was recorded on the Nicolet 6,700 spectrometer with an MCT detector. For CO adsorption analysis, the sample was reduced *in situ* at 300°C H_2_ (20 mL/min) for 1 h, scanned in Ar for 30 min, and then cooled to room temperature for CO adsorption for 30 min. Then Ar scanning was performed again to remove the adsorbed CO substance for 30 min, and the spectrum was collected. The experimental procedure of For *in-situ* FTIR spectroscopy of CO_2_ hydrogenation, the catalyst was reduced *in situ* in H_2_ (20 mL/min) at 300°C for 1 h, then purged with He for 30 min to remove the physically adsorbed H_2_, and then cooled to 40°C to collect the background spectrum. Finally, He was replaced with a mixture of raw materials (CO_2_:H_2_:N_2_ = 24:72:4), and the process of the intermediate product changing with time was recorded, and the infrared spectrum with a resolution of 4 cm^−1^ was obtained.

### 2.3 Catalytic activity tests

Methanol synthesis via CO_2_ hydrogenation was carried out in a high-pressure continuous flow fixed-bed reactor. Before the reaction of CO_2_ hydrogenation to methanol, 0.5 g catalyst mixed with 2.0 g quartz sand (both 20–40 mesh) was loaded into a stainless steel reactor tube with an inner diameter of 10 mm. Firstly, the catalyst was reduced by H_2_ (50 mL/min) at 300°C for 4 h. Then, the reactor was cooled to 200°C, and the feed gas (24% CO_2_, 72% H_2_, and 4% N_2_) with the gas hourly space velocity (GHSV) of 9,000 mL⋅g_cat_^−1^⋅h^−1^ was introduced in the reactor that was pressurized to 2.5 MPa. All pipelines and valves were heated to 140°C by a heating belt to prevent condensation of gas products. The products were quantitatively analyzed online by a Shimadzu GC-2014 gas chromatograph equipped with a thermal conductivity detector (TCD) and a flame ionization detector (FID). The CO_2_ conversion rate (X_CO2_), selectivity of CH_3_OH(S_CH3OH_) and space-time yields of CH_3_OH(STY_CH3OH_, g⋅kg_cat_^−1^⋅h^−1^) were defined as the following Eqs 4–6:

$$X_{CO2}=\frac{{\left[CO\right.\left.2\right]}_{in}-{\left[CO\right.\left.2\right]}_{out}}{{\left[CO\right.\left.2\right]}_{in}}\times 100\%$$ (4)

$$S_{CH3OH}=\frac{{\left[CH\right.3\left.OH\right]}_{out}}{{\left[CO\right.\left.2\right]}_{in}-{\left[CO\right.\left.2\right]}_{out}}\times 100\%$$ (5)

$${STY}_{CH3OH}=\frac{F_{CO2,in}\times X_{CO2}\times S_{CH3OH}\times M_{CH3OH}}{W_{cat}}$$ (6)

Where, [CO_2_]_in_ and [CO_2_]_out_ represent the input and output of carbon dioxide (mol), [CH_3_OH]_out_ represents the output of methanol (mol), F_CO2_,_in_ represents the molar input flow of carbon dioxide (mol⋅h^−1^), and W_cat_ represents the mass of catalyst (g). M_CH3OH_ represents the molar mass of methanol (g⋅mol^−1^).

## 3 Results and discussion headings

### 3.1 Physicochemical properties of catalysts

**FIGURE 1:**
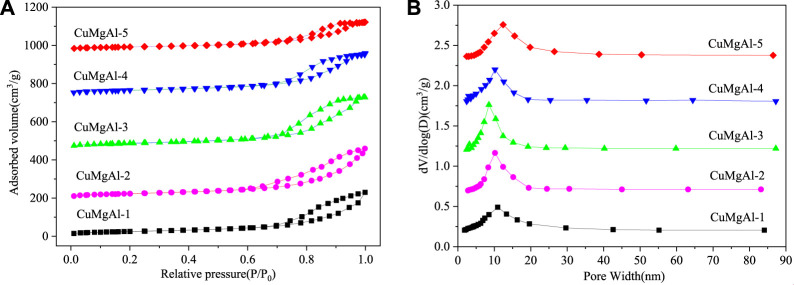
N_2_ physical adsorption-desorption of CuMgAl-x catalysts: **(A)** N_2_ adsorption isotherm; **(B)** Pore size distribution curves.

The N_2_ adsorption-desorption isotherm and pore size distribution of CuMgAl-x catalysts are shown in Figure 1. Figure 1A showed that all samples had typical type IV adsorption isotherms with different hysteresis curves, indicating that CuMgAl-x catalysts had a typical mesoporous structure (Zeng et al., 2013). CuMgAl-1 and CuMgAl-2 catalysts exhibited typical H3-type hysteretic loops, which were related to the porosity and slit caused by the aggregation of lamellar particles. In addition, H2-type hysteretic loops can be observed on CuMgAl-4 and CuMgAl-5 samples, which were often associated with complex and interconnected pore structures (León et al., 2010). However, the isotherms of CuMgAl-3 samples showed an intermediate shape between CuMgAl-2 and CuMgAl-4. The pore structure of the sample changed obviously with the increase of the Mg/Al molar ratio. The chemical composition and structural characteristics of the samples are listed in Table 1. CuMgAl-3 had the highest S_BET_ of 192.3 m^2^g^−1^ among all samples, and the S_BET_ value decreased significantly with the further increase of the Mg/Al ratio. On the whole, the change of pore diameter was opposite to S_BET_ (Figure 1B). The variation of total specific surface area, pore volume, and average pore size with Mg/Al molar ratio may be related to the difference in pore structure.

**TABLE 1:** Textural Properties of CuMgAl-x catalysts.

Catalysts	Cu loadings^a^ (wt.%)	Mg/Al molar ratio^a^	Surface area^b^ (m^2^g^−1^)	Pore volume^b^ (cm^3^g−^1^)	Pore size^b^ (nm)
CuMgAl-1	10.2	1.1	130.4	0.345	10.4
CuMgA-2	10.4	2.2	151.4	0.352	9.7
CuMgA-3	10.1	3.0	192.3	0.384	7.8
CuMgA-4	10.5	4.1	167.5	0.367	9.1
CuMgA-5	10.7	5.3	123.2	0.323	11.6

^a^ Measured by ICP-OES.

^b^ The surface area, pore volume and pore size of the catalysts were deter-mined by the N_2_ adsorption-desorption experiment.

**FIGURE 2:**
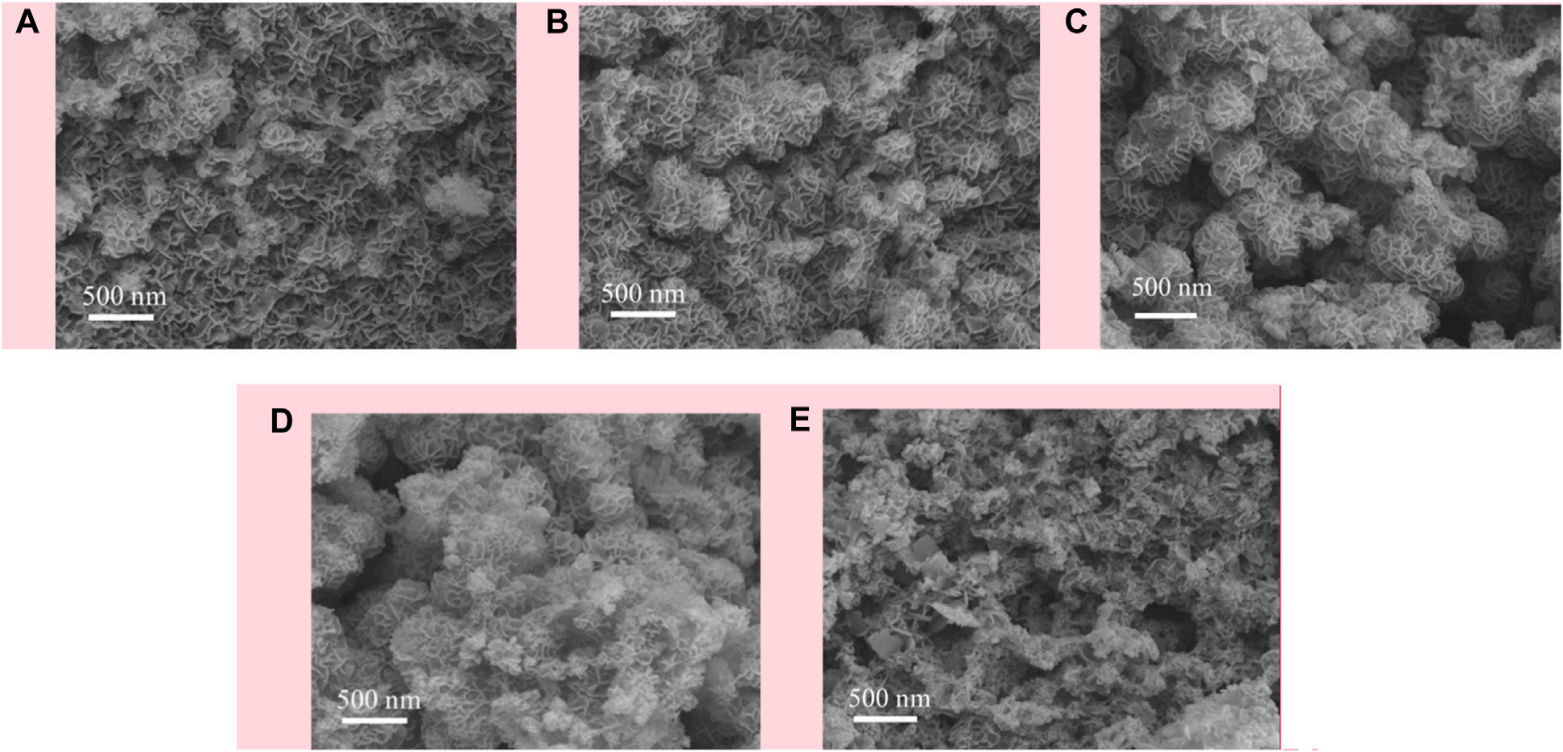
SEM images of the CuMgAl-x catalysts: **(A)** CuMgAl-1, **(B)** CuMgA-2,**(C)** CuMgA-3, **(D)** CuMgA-4, **(E)** CuMgAl-5.

The morphology of calcined CuMgAl-x catalysts was characterized by scanning electron microscope (SEM), and the results are shown in Figure 2. Figure 2A shows the SEM images of the CuMgAl-1 catalyst, the surface of the sample was inlaid with many irregularly shaped nanosheets. In contrast, the existence forms of nanosheets on CuMgAl-2 and CuMgAl-3 samples were quite different, which was reflected in the cross of nanosheets and the formation of flower-like spheres, which was related to the formation of pores (Shao et al., 2020). It can be found that the flower-like sphere structure formed by nanosheets on CuMgAl-3 samples was more regular, which was conducive to the exposure of pores. When the Mg/Al ratio increased to 4 and 5, the flower-shaped spheres gradually disappeared and formed a more complex pore structure. These results were consistent with BET results, indicating that the morphology of samples varied with the Mg/Al ratio.

**FIGURE 3:**
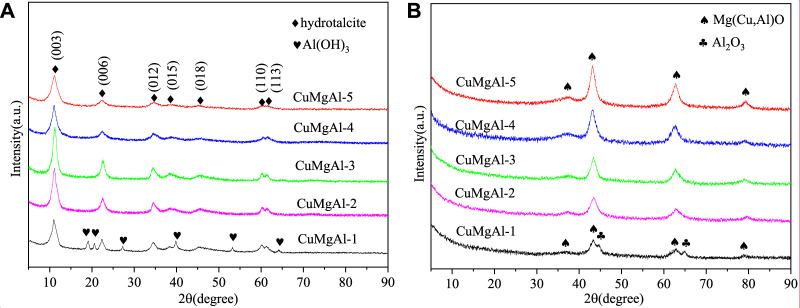
XRD patterns of CuMgAl-x catalysts: **(A)** before calcination, **(B)** after calcination.

The CuMgAl-x catalysts were characterized by X-ray diffraction (XRD) to detect the crystallinity of the phases, and the results are shown in Figure 3. Figure 3A shows the XRD patterns of the CuMgAl-x precursors. The X-ray characteristic diffraction peaks at 2θ = 11.4°, 22.6°, 34.8°, 38.8°, 45.3°, 60.4° and 61.7° were respectively attributed to the (003), (006), (012), (015), (018), (110) and (113) crystal planes of Mg-Al hydrotalcite structure (JCPDS35-0965), indicating that the hydrotalcite structure was formed successfully in the CuMgAl-x catalysts precursors (Shao et al., 2019). The peak strength of the hydrotalcite crystal plane of CuMgAl-x catalysts precursors gradually increased with the decrease of the Mg/Al molar ratio and reached the maximum value when Mg/Al = 3, which means that CuMgAl-3 had the best crystallinity. Since the charge density of Al^3+^ was higher than that of Mg^2+^, the increase of Al^3+^ contents increased the charge density on the layer, resulting in the regular layered structure and strong interlayer interaction of CuMgAl-3 (Shi et al., 2021). The crystallinity of the sample gradually decreases with the further decrease the Mg/Al ratio, which may be caused by the decrease of stability of hydrotalcite caused by excessive Al. The diffraction peak of aluminum hydroxide was only observed in the CuMgAl-1 sample, which may be due to the large amount of Al^3+^ can not enter the CuMgAl hydrotalcite structure and precipitated in the form of Al(OH)_3_.

Figure 3B shows the XRD patterns of the calcined CuMgAl-x catalysts at 500°C. After the calcination of precursors, the hydrotalcite structure disappeared in the XRD patterns, indicating that the hydrotalcite structure was destroyed by high-temperature calcination. The X-ray characteristic diffraction peaks at 2θ = 36.5°, 43.1°, 62.7°, and 78.9° correspond to the (111), (200), (220) and (222) crystal planes of MgO (Huang et al., 2015), respectively, and the characteristic peak intensity of MgO increased with the increase of Mg/Al molar ratio. The X-ray diffraction pattern of the CuMgAl-1 calcined sample showed some peaks corresponding to Al_2_O_3_, which was consistent with the diffraction pattern for the analysis of hydrotalcite precursors. It seemed that after the calcination process, some of the Al^3+^ in the laminates became part of the lattice while others remained separated, resulting in the production of aluminum oxide. However, as the Mg/Al molar ratio increases, no characteristic peaks associated with CuO, CuAl_2_O_4_ spinel, and Al_2_O_3_ are detected, possibly because Cu^2+^ combined with Al^3+^ into the MgO structure to form Mg(Cu, Al)O solid solutions, indicating that CuO has good dispersion on Mg(Al)O carriers (Li et al., 2016).

**FIGURE 4:**
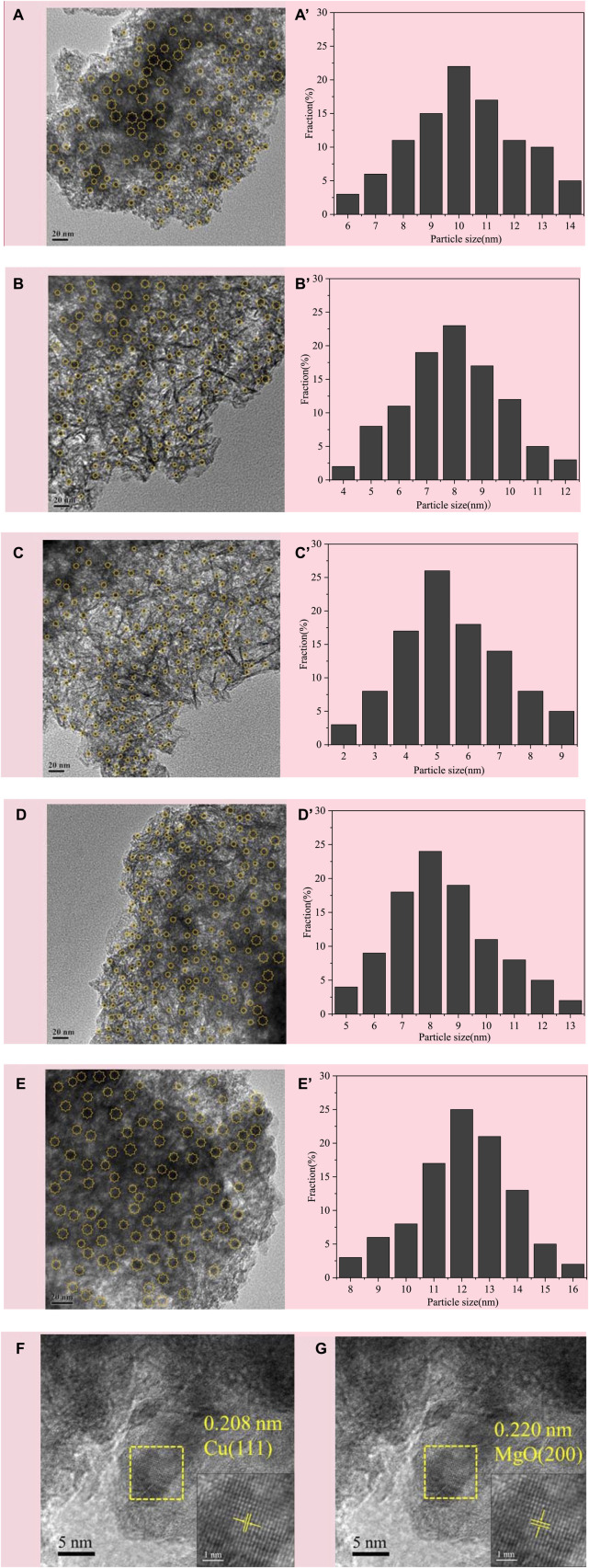
TEM images of the reduced CuMgAl-x catalysts, **(A)** CuMgAl-1, **(B)** CuMgA-2, **(C)** CuMgA-3, **(D)** CuMgA-4, **(E)** CuMgAl-5, **(A′–E′)** corresponding Cu particle size distribution, **(F–G)** HR-TEM images of CuMgA-3.

The morphology and Cu particle size distribution of CuMgAl-x catalysts after reduction were observed by transmission electron microscopy (TEM), as shown in Figure 4. The average Cu particle sizes for each sample are shown in Table 2. It was evident that CuMgAl-3 exhibited a minimum mean particle size of 5.57 nm and a good distribution of Cu particles due to the hydrotalcite structure with high crystallinity, which was conducive to the reduction of Cu particles. In summary, the average particle size of all samples was sorted as follows: CuMgAl-5 (12.02 nm)>CuMgAl-1 (10.13 nm)>CuMgAl-4 (8.25 nm)>CuMgAl-2 (7.95 nm)>CuMgAl-3 (5.57 nm). It was worth noting that neither too high nor too low Mg/Al ratio can obtain satisfactory particle distribution and small particle size of Cu, which indicates that the Mg/Al ratio has a significant effect on the size and distribution of Cu metal particles. The dispersion of active components can be further analyzed by EDX characterization. As shown in Supplementary Figure S1, Cu, Mg, Al, and O elements were uniformly dispersed in the CuMgAl-3 catalyst. Figures 4F, G showed the HRTEM images of CuMgAl-3, in which it can be observed that the lattice fringe of Cu(111) and MgO(220) were in the same region and presented a state of mutual combination, which proved that there is a strong interaction between Cu and MgO (Chen et al., 2024).

**TABLE 2:** Average Cu size, Cu dispersions and Cu specific surface area of CuMgAl-x catalysts.

Catalysts	d_Cu_ ^a^/(nm)	D_Cu_ ^b^/(%)	S_Cu_ ^b^/(m^2^g−^1^)
CuMgA-1	10.13	22.8	22.7
CuMgA-2	7.95	26.2	30.5
CuMgA-3	5.57	31.5	33.3
CuMgA-4	8.25	24.6	28.2
CuMgA-5	12.02	19.1	18.6

^a^ The size of Cu particles (d_Cu_) on the reduced Cu-based catalyst was calculated by TEM.

^b^ The dispersion (D_Cu_) and surface area (S_Cu_) of Cu particles on the catalyst surface were determined by the N_2_O chemisorption method.

**FIGURE 5:**
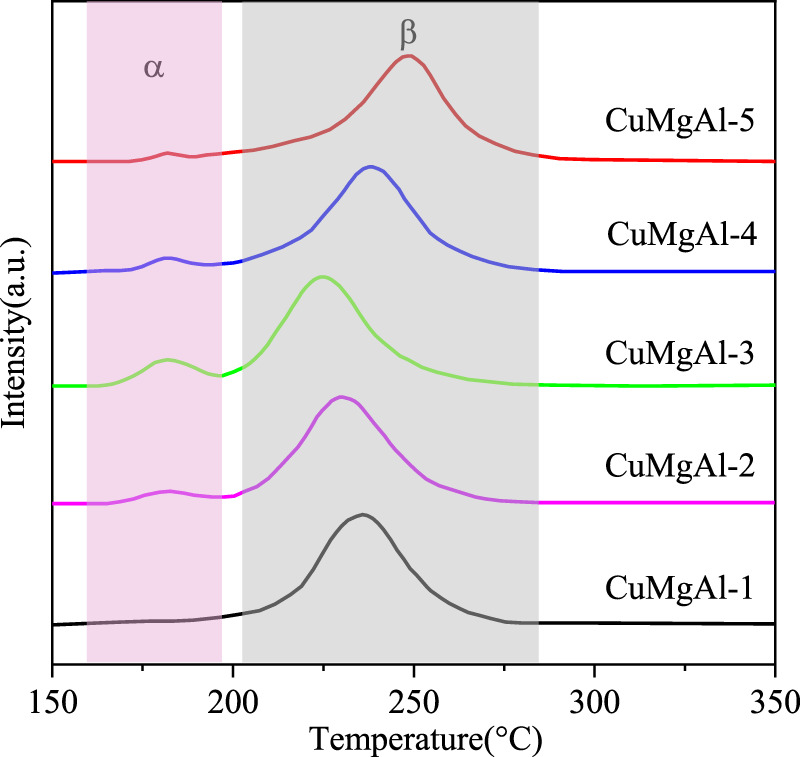
H_2_-TPR curves of the calcined CuMgAl-x catalysts The basic property of the reduced CuMgAl-x catalysts was characterized by CO_2_-TPD, and the results are shown in Figure 6 and Table 3. Desorption curves of all catalysts can be deconvoluted into three Gaussian peaks. The desorption peaks at about 201°C belong to weak basic sites (α peak), the desorption peaks at 298°C belong to moderate strong basic sites (β peak), and the desorption peaks above 441°C belong to strong basic sites (γ peak) (Liu et al., 2013). The weak basic sites are related to the hydroxyl group on the catalyst surface, the moderate basic sites are attributed to the Mg-O pairs, and the strong basic sites are related to the low coordination unsaturated O^2-^ ions (León et al., 2010). It can be observed that the desorption peak shifted to higher temperatures as the Mg/Al ratio increased from 1 to 3. In addition, the total number of basic sites and proportion of moderate-strong basic sites in catalysts also increased, which was conducive to CO_2_ adsorption and further hydrogenation to methanol (Table 2). But, when the ratio of Mg/Al increased from 3 to 5, the number and intensity of basic sites decreased. This may be due to the higher Mg/Al ratio resulting in partial collapse of the hydrotalcite during the synthesis process and reduced the crystallinity (Huang et al., 2015), which was consistent with XRD analysis.

The reduction performance of CuMgAl-x catalysts was analyzed through the characterization of hydrogen temperature programmed reduction (H_2_-TPR). As shown in Figure 5, all samples exhibited two reduction peaks, where the α peak at around 181°C was attributed to the reduction of highly dispersed CuO particles, while the β peak at higher temperatures (>210°C) was attributed to the reduction of bulk CuO interacting with MgO or Al_2_O_3_ (Chen et al., 2024). Obviously, CuO species on the surface of CuMgAl-3 catalyst had better dispersion than other catalysts. When the Mg/Al molar ratio increased from 1 to 3, the reduction temperature of the β peak decreased from 235°C to 224°C, which may be due to the strong interaction between CuO and MgO, which weaken the strength of the Cu-O bond. However, as the Mg/Al molar ratio increased from 3 to 5, the reduction temperature of CuO increased again, which was related to the accumulation of CuO.

**FIGURE 6:**
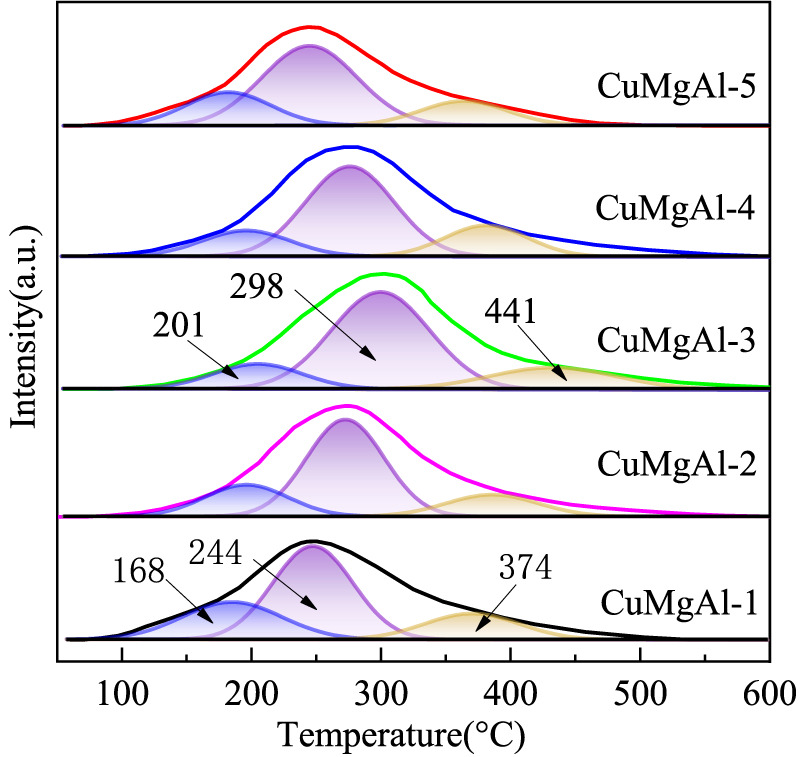
CO_2_-TPD curves of the reduced CuMgAl-x catalysts.

**TABLE 3:** The distribution of basic sites over CuMgAl-x catalysts.

Catalysts	Temperature (°C)			Total basic sites (μmol/g)	Peak area fraction (%)	
	Site α	Site β	Site γ		Site α	Site β+γ
CuMgA-1	168	244	374	223	23.4	76.6
CuMgA-2	187	269	397	248	16.2	83.8
CuMgA-3	201	298	441	261	14.1	85.9
CuMgA-4	191	275	388	233	18.3	81.7
CuMgA-5	176	244	369	215	27.3	72.7

**FIGURE 7:**
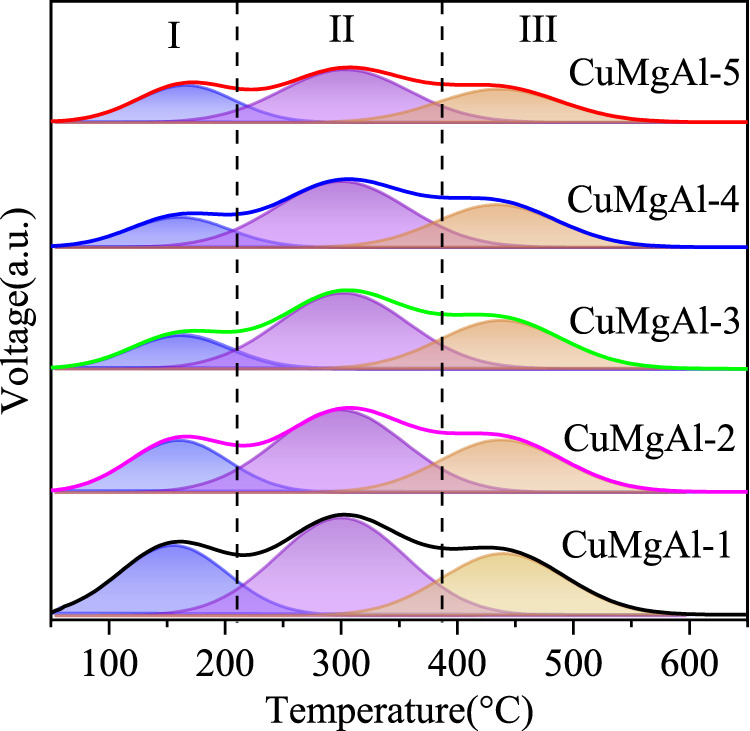
NH_3_-TPD curves of the CuMgAl-x catalysts.

It is well known that the performace of CO_2_ hydrogenation to methanol is also closely related to the acidity of the catalyst surface (Zhou et al., 2021), which can be detected by NH_3_ temperature-programmed desorption (NH_3_-TPD). The NH_3_-TPD profiles of CuMgAl-x catalysts are illustrated in Figure 7. Using the Gaussian curve fitting method, the peaks are also deconvoluted to obtain the semiquantitative analysis. The calculated results are listed in Table 4. The desorption peaks at about 155°C belong to weak acid sites (Ⅰ peak), the desorption peaks at 301°C belong to medium acid sites (Ⅱ peak), and the desorption peaks above 446°C belong to strong acid sites (Ⅲ peak), which should be attributed to the Lewis acidity of MgO-Al_2_O_3_ mixed oxide (Xia et al., 2016). It is evident that when the Mg/Al molar ratio rises, the amount of total acidic sites of CuMgAl-x catalysts falls noticeably, and the CuMgAl-1 catalyst with the lowest Mg concentration showed the highest amount of total acidic sites. The explanation for this is that the presence of MgO can lessen the acidity of catalysts. Moreover, Table 4 shows that the proportion of acid sites at the two high temperature peaks (II + III) of CuMgAl-3 is 82.4%, which is higher than other catalysts.

**TABLE 4:** The distribution of acidic sites over CuMgAl-x catalysts.

Catalysts	Temperature (°C)			Total acidic sites (μmol/g)	Peak area fraction (%)	
	Ⅰ	Ⅱ	Ⅲ		Ⅰ	Ⅱ+Ⅲ
CuMgA-1	155	301	446	192	27.3	72.7
CuMgA-2	156	298	448	176	22.5	77.5
CuMgA-3	158	301	447	163	17.6	82.4
CuMgA-4	157	300	444	152	20.8	79.2
CuMgA-5	159	303	446	133	26.2	73.8

**FIGURE 8:**
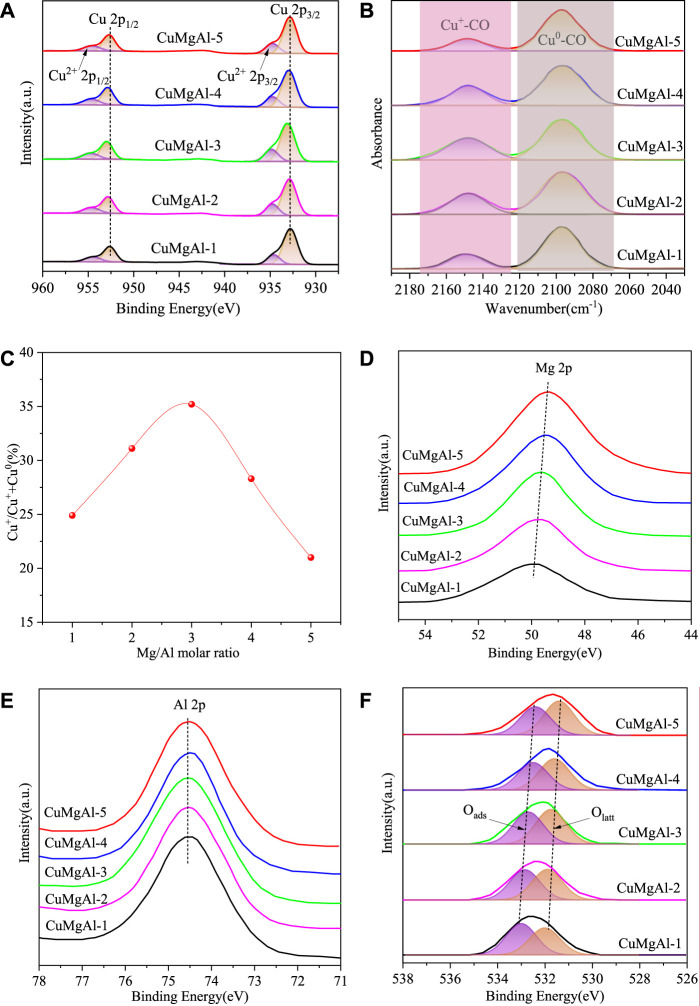
**(A)** XPS spectra in the region of Cu 2p, **(B)** Infrared spectra of CO adsorption on the reduced CuMgAl-x catalysts, **(C)** The relationship between the ratio of Cu^+^/(Cu^+^+Cu^0^) and the molar ratio of Mg/Al, **(D–F)** XPS spectra in the region of Mg 2p, Al 2p and O 1s.

XPS analysis of all catalysts was carried out to investigate the important influence of catalyst surface Cu state on the catalytic behavior. Figure 8A shows the XPS spectra of all CuMgAl-x catalysts after reduction. In the Cu 2p spectrum, the characteristic peaks at 932.8 and 952.5eV corresponded to the reduced Cu^0^/Cu^+^, and the characteristic peaks at 934.8 and 954.7 corresponded to the Cu^2+^ species (Song et al., 2023b). The binding energy of Cu 2p_3/2_ of CuMgAl-x catalyst (932.8–933.1 eV) was higher than the standard value (932.6 eV), which indicated the existence of electron transfer from Cu to MgO, forming a metal-support strong interaction (MSI) (Wang et al., 2023b). In order to further study the chemical state on the surface of the reduction catalyst, the distribution of Cu species was further studied by *in-situ* infrared spectroscopy using CO as the probe molecule of the irreversible adsorption reaction, and the results were shown in Figure 8B. After CO adsorption and He purification, the physically adsorbed CO disappeared, while the chemisorbed CO bands remained near 2,148 cm^−1^ and 2096 cm^−1^, which were attributed to the linear stretching of Cu^+^-CO and Cu^0^-CO species (Nielsen et al., 2021), respectively. This indicated that copper oxide was reduced to Cu^+^/Cu^0^ pairs rather than fully reduced to Cu^0^ species under the reduction conditions in this study. Supplementary Figure S2 displays the Cu LMM Auger transition spectra of the reduced CuMgAl-x catalysts. The characteristic peaks near 912.7 eV and 918.5 eV are responsible for the signals of Cu^+^ and Cu^0^, respectively. The percentage of Cu^+^ and Cu^0^ content can be estimated by integrating the two characteristic peaks (Lu et al., 2019; Zhao et al., 2021). As shown in Figure 8C, a volcanic trend was presented between the ratio of Cu^+^/(Cu^+^+Cu^0^) and the molar ratio of Mg/Al. Among all the samples, CuMgAl-3 reached the highest value of 36.7%. This difference allowed us to relate the interaction between CuO and MgO, and the stronger interaction was conducive to the formation of more Cu^+^ species in the Cu-MgO interface, which has been confirmed by many researchers (Wang et al., 2023a; Chen et al., 2023; Zhang et al., 2023). Figures 8D–F) showed the XPS spectra of Mg 2p, Al 2p and O 1s, respectively. With the increase of Mg content, Mg 2p gradually moved to the direction of low binding energy, and Al 2p basically remained unchanged. In the O 1s spectrum, two characteristic peaks were observed at 531.9 and 533.0 eV, belonging to lattice oxygen (O_latt_) and adsorbed oxygen (O_ads_), respectively (Zhang et al., 2023). Obviously, with the increase of Mg content, O_latt_ and O_ads_ moved to the direction of low binding energy, which was caused by the strong interaction between Cu and MgO (Chen. et al., 2024; Sha et al., 2021).

### 3.2 Catalytic activity tests

**FIGURE 9:**
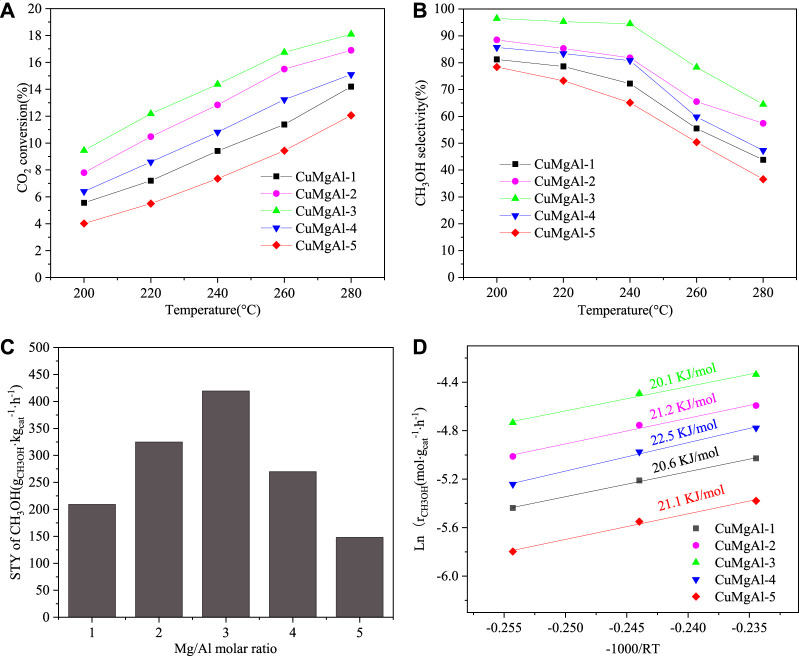
**(A)** CO_2_ conversion, **(B)** CH_3_OH selectivity, **(C)** The relationship between STY of CH_3_OH and Mg/Al molar ration, **(D)** Arrhenius plots for CuMgAl-x catalyst. The catalytic performance of CuMgAl-3 catalyst over time. Reaction conditions: 240°C, 2.5 Mpa and 9,000 mL⋅g_cat_^−1^⋅h^−1^, CO_2_/H_2_/N_2_ (molar ratio = 24:72:4)

Figure 9 shows the catalytic performance of CO_2_ hydrogenation to methanol on the prepared CuMgAl-x catalysts. It can be noted in Figure 9A that the conversion of CO_2_ increased with the increase in reaction temperature, which indicated that high tem-perature was conducive to the activation and conversion of CO_2_. It can be found in Figure 9B that the selectivity of CH_3_OH decreased with the increase in temperature, which was contrary to the change trend of CO_2_ conversion. In fact, there are two important competitive reactions in CO_2_ hydrogenation to methanol. The first one is the methanol synthesis, and the second one is the reverse water gas shift (RWGS) reaction (Singh et al., 2021; Stangeland et al., 2021). The equilibrium of these reactions can be described as Eqs 7, 8:

$${\text{CO}}_{2}+3H_{2}↔{\text{CH}}_{3}\text{OH}+H_{2}O\;\Delta H=‐49.5{\text{kJmol}}^{‐1}$$ (7)

$${\text{CO}}_{2}+H_{2}↔\text{CO}+H_{2}O\;\Delta H=+41.2{\text{kJmol}}^{‐1}$$ (8)

As shown in reaction (7), methanol synthesis was an exothermic reversible reaction and its equilibrium constant decreased with the increase in reaction temperature. In addition, compared with the RWGS reaction (8), the methanol synthesis reaction had lower apparent activation energy. Therefore, in the whole temperature range, the selectivity of CH_3_OH decreased with the increase in reaction temperature. As shown in Figure 9C, according to the data of CO_2_ conversion and methanol selectivity, the relationship between space-time yield of methanol (STY_CH3OH_) and Mg/Al molar ratio was obtained with a volcanic relationship, which indicated that the Mg/Al molar ratio had a greater influence on the performance of CuMgAl-x catalysts. The order of STY_CH3OH_ of all catalysts at this temperature was as follows: CuMgAl-3 (418.9 g⋅kg_cat._^−1^⋅h^−1^)>CuMgAl-2 (323.8 g⋅kg_cat._^−1^⋅h^−1^)>CuMgAl-4 (269.4 g⋅kg_cat._^−1^⋅h^−1^)>CuMgAl-1 (209.6 g⋅kg_cat._^−1^⋅h^−1^)>CuMgAl-5 (147.6 g⋅kg_cat._^−1^⋅h^−1^). The catalytic performance of all catalysts was measured under the conditions of temperature(T) = 240°C, pressure(P) = 2.5 MPa, WHSV = 9,000 mL⋅g_cat_^−1^⋅h^−1^, CO_2_/H_2_/N_2_ (molar ratio) = 24:72:4. The apparent activation energy (E) was calculated by the Arrhenius equation (Lu et al., 2018; Zhang et al., 2024a), as shown in Figure 9D. Among all catalysts, CuMgAl-3 showed the lowest apparent activation energy of 20.1 kJ/mol.

The operational stability of catalysts is one of the key issues in catalyst development. The catalytic performance of the CuMgAl-3 catalyst changes over time, as shown in Supplementary Figure S3. Both CO_2_ conversion and methanol selectivity showed good stability during the catalysis test for 150 h, indicating that the active site in the CuMgAl-3 catalyst remained stable. As shown in Supplementary Figure S4, the spent-CuMgAl-3 catalyst was characterized by XRD and compared with the fresh-CuMgAl-3 catalyst. It can be seen from the results that the XRD pattern of the spent-CuMgAl-3 catalyst was similar to that of the fresh-CuMgAl-3 catalyst, and no obvious characteristic peak of metal Cu was found, indicating that Cu particles were well dispersed after the reaction. As shown in Supplementary Figure S5, the TEM characterization of the used CuMgAl-3 catalyst showed that the Cu particle size after the reaction (7.48 nm) was slightly increased compared with that before the reaction (5.57 nm), and there was no obvious sintering, indicating that the Cu particles in CuMgAl-3 catalyst still had good dispersion after the reaction.

**FIGURE 10:**
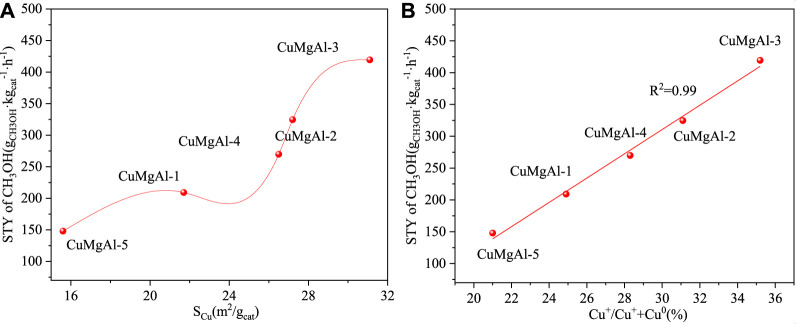
**(A)** The relationship between STY of CH_3_OH and S_Cu_, **(B)** The relationship between STY of CH_3_OH and the ratio of Cu^+^/(Cu^+^+Cu^0^).

The above results indicated that a suitable molar ratio of Mg/Al was beneficial to improve the catalytic performance of CuMgAl-x catalyst for CO_2_ hydrogenation to methanol. CuMgAl-3 catalyst with Mg/Al molar ratio of 3 had the maximum CO_2_ conversion (14.3%) and methanol selectivity (94.5%) at 240°C and 2.5 MPa. CuMgAl-3 catalyst with the best performance had the highest Cu dispersion (32.4%) and the largest Cu surface area (S_Cu_, 31.1 m^2^/g), exposing the most Cu species, which was conducive to the interaction of Cu particles with MgO and formed more Cu-MgO active interfaces. As shown in Figure 10A, there was a positive correlation between STY_CH3OH_ and S_Cu_. The high S_Cu_ provided more active sites for the adsorption and activation of CO_2_ and H_2_, thus obtaining higher catalytic activity (Dharmalingam et al., 2023). However, the relationship between STY_CH3OH_ and S_Cu_ was not linear, which proved that not only does the S_Cu_ have an effect on catalytic activity, but other factors also have an effect on catalytic activity. In addition, the electronic state of Cu also affected the catalytic performance of Cu-based catalysts (Zheng et al., 2023). It can be seen from Figure 8 that both Cu^0^ and Cu^+^ existed on the CuMgAl-x catalyst, and the ratio of Cu^+^/(Cu^+^+Cu^0^) can be adjusted by changing the Mg/Al molar ratio. Generally, Cu^0^ is exposed to the surface of the catalyst and played a fundamental role in the adsorption and activation of H_2_, while Cu^+^ produced by the strong interaction between Cu and the carrier plays a positive role in the adsorption of CO, which effectively improves the selectivity of methanol. (Samson et al., 2014; Song et al., 2023a). Figure 10B shows the relationship between STY_CH3OH_ and the ratio of Cu^+^/(Cu^+^ +Cu^0^), and the relationship between the two was linear, which indicated that Cu^+^ had a greater influence on the performance of the catalyst.

In order to further analyze the catalytic performance of the CuMgAl-3 catalyst, CuMgAl-3 catalyst were compared with some typical catalysts for CO_2_ hydrogenation to methanol. Based on the STY_CH3OH_, the results were listed in Supplementary Table S1. The catalytic performance of the CuMgAl-3 catalyst was better than that of traditional Cu-based catalysts (Lei et al., 2016; Xiao et al., 2017; Chen et al., 2022) and the catalysts containing Mg and Al elements at the same time (Ren et al., 2015; Fang et al., 2019a; Fang et al., 2019b; Cored et al., 2022). The STY_CH3OH_ of CU-0.5-300 (Liu et al., 2020) and Pd/In_2_O_3_ (Rui et al., 2017) was higher than that of CuMgAl-3, mainly because the activity tests of these catalysts were performed at higher GHSV, pressure, or temperature. In particular, the increase in the GHSV can significantly increase the methanol production capacity, resulting in higher STY_CH3OH_. In summary, CuMgAl-3 has excellent catalytic performance with the advantages of low cost, green environmental protection, and easy industrial production, so it can be used as an ideal catalyst for methanol synthesis.

### 3.3 Reaction pathway and structure-performance relationship

**FIGURE 11:**
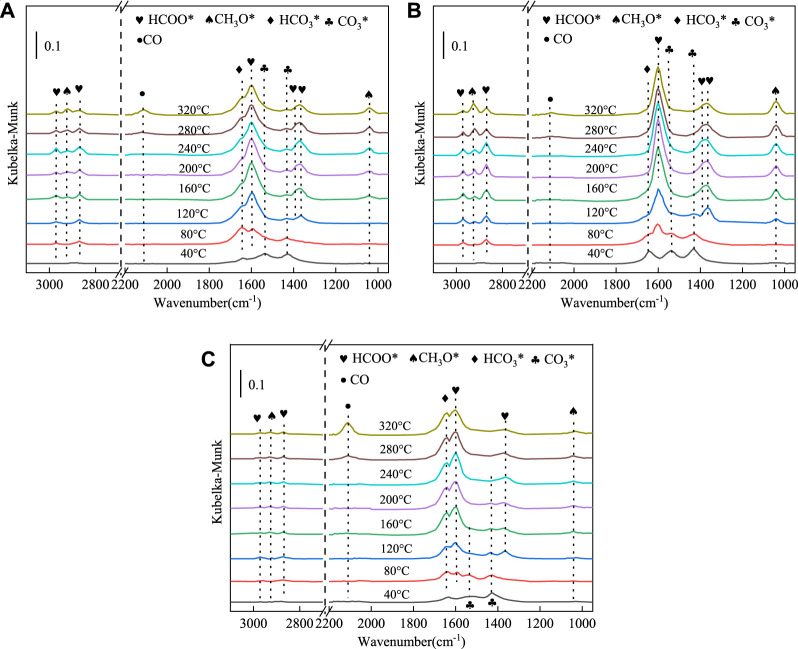
*In situ* FTIR spectra of CO_2_ hydrogenation reaction over **(A)** CuMgAl-1, **(B)** CuMgAl-3, **(C)** CuMgAl-5 catalysts in the test temperature range from 40°C to 320°C. (0.1 Mpa, H_2_/CO_2_/N_2_ = 72:24:4)

*In situ* DRIFTS experiments were conducted on CuMgAl-1, CuMgAl-3 and CuMgAl-5 catalysts using CO_2_ + H_2_ as reactants to explore the possible mechanism of CO_2_ hydrogenation. Figure 11 shows the transient evolution of major surface substances during CO_2_ hydrogenation of these catalysts at 40°C–320°C. For the CuMgAl-1 catalyst (Figure 11A), three characteristic peaks were found at 1431,1538 and 1,646 cm^−1^ are attributed to carbonate (CO_3_*) and bicarbonate (HCO_3_*) at 40°C, and no other characteristic peaks were found at this temperature (Hartadi et al., 2016). When the temperature rises further to 120°C, the characteristic peak intensity of formate (the peaks at 1,601, 1,362, 2,872 and 2,968 cm^−1^attributed to the symmetric vibration of ν_s_ (OCO), the symmetric OCO stretching vibrations ν_s_ (OCO), the CH stretching vibrations ν(CH), the CH bending δ(CH) and asymmetric OCO stretching vibration ν_as_ (OCO), respectively) increases rapidly, but the characteristic peak of the methoxy species (the peaks at 2,928 and 1,040 cm^−1^ attributed to the asymmetric CH_3_ stretching vibration ν_as_ (CH_3_) and the OCO stretching vibrations ν(CO), respectively) does not appear until 160°C (Yan et al., 2022; Wang et al., 2023b; Tian et al., 2023). When the temperature gradually increases to 240°C, the conversion of formate (HCOO*) to CH_3_O* substance was promoted, which is characterized by a gradually stronger signal of CH_3_O* and a gradually weaker band of HCOO*. At the same time, it was found that the strength of bicarbonate (HCO_3_*) reached the maximum when the temperature rose to 120°C, but there was no obvious change in strength when the temperature continued to rise, indicating that the ability of HCO_3_* to convert into HCOO* on CuMgAl-1 was weak. In addition, the characteristic peak of CO was found in 2013 cm^−1^, indicating the existence of RGWS reaction, and high temperature was conducive to the generation of CO (Kattel et al., 2016).

For the CuMgAl-3 catalyst (Figure 11B), the signal strength of CO_3_* and HCO_3_* was stronger than that of CuMgAl-1 at 40°C, indicating that the CuMgAl-3 had stronger CO_2_ adsorption capacity, which was consistent with the conclusion in Figure 6. With the increase of temperature, the conversion of CO_3_* species to HCOO* species can still be found, but the signal strength of the CH_3_O* group on CuMgAl-3 was significantly greater than that on CuMgAl-1, which was consistent with less CO_3_* accumulation on CuMgAl-3. In addition, the HCO_3_* characteristic peak on CuMgAl-3 completely disappeared at 200°C. This phenomenon indicated that CO_3_* and HCO_3_^−^ species on CuMgAl-3 were more easily converted to formates at low temperatures, which was conducive to further conversion to methanol, which may relate to the stronger Cu-MgO interaction on CuMgAl-3 catalyst. It can be seen from the H_2_-TPD (Supplementary Figure S6) results that CuMgAl-3 had the largest H_2_ desorption peak at low-temperature, indicating that it had the best H_2_ activation capacity because the strong Cu-MgO interaction was conducive to the formation of highly dispersed Cu nanoparticles that promote H_2_ dissociation, which will promote the hydrogenation of intermediate species CO_3_* and HCO_3_^−^ to HCOO*. At the same time, it was found that the accumulation of CO in CuMgAl-3 at high-temperature was smaller than that in CuMgAl-1, which was because the formation of more Cu^+^ enhances the adsorption of CO, thus improving the selectivity of methanol (Yao et al., 2019). For the CuMgAl-5 catalyst (Figure 11C), the HCOO* species and CH_3_O* group strength were lower than CuMgAl-1 and CuMgAl-3 during temperature rise, and the CO strength was the highest at high-temperature, indicating that excessive Mg/Al has a negative effect on the catalytic performance.

**FIGURE 12:**
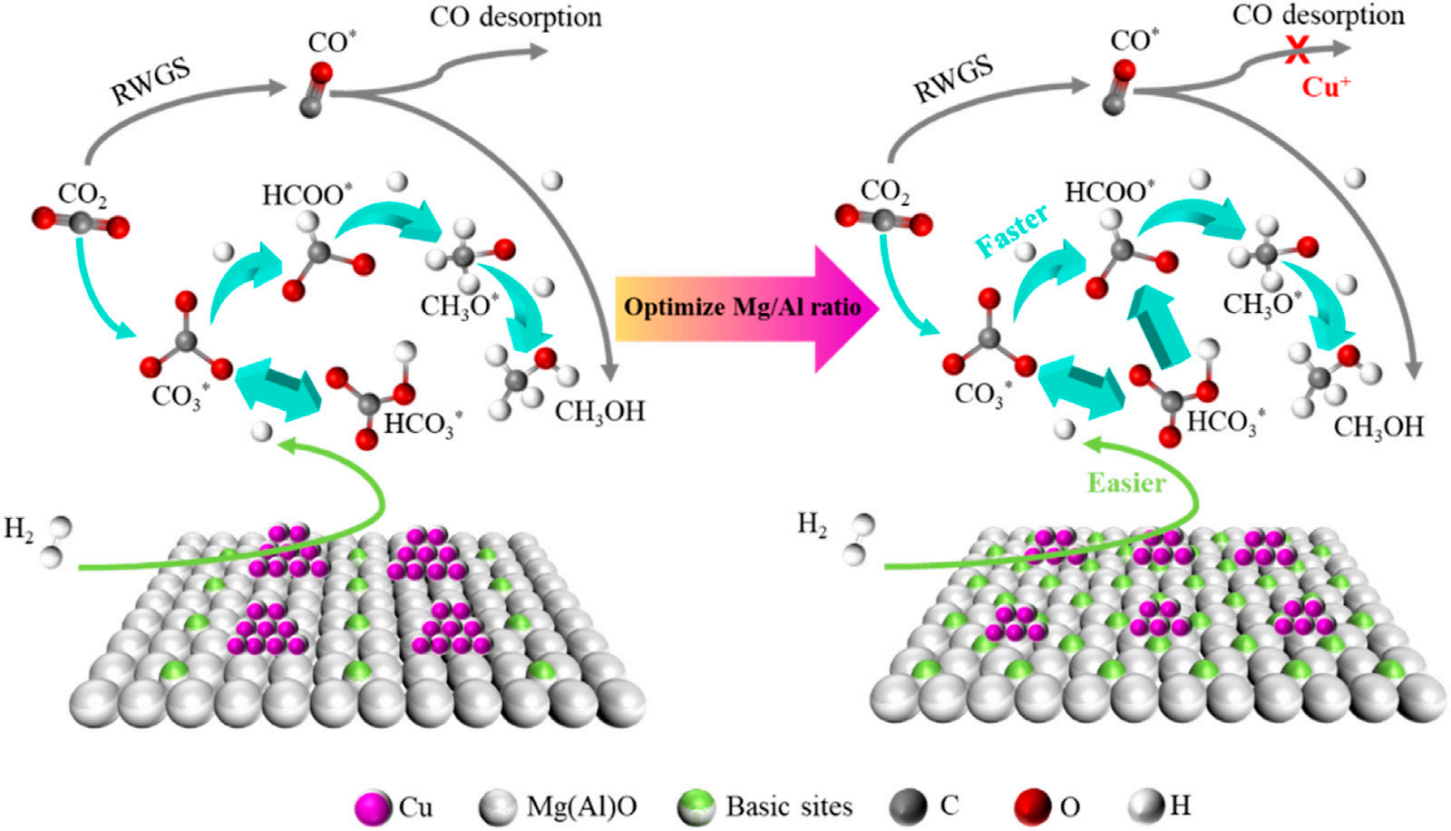
Mechanism comparison of CuMgAl-x catalysts for CO_2_ hydrogenation to methanol before and after the optimization of Mg/Al ratio.

Based on the above discussion, there is no doubt that the interaction between active Cu and MgO on CuMgAl-3 catalyst plays an irreplaceable role in the hydrogenation of CO_2_ to the methanol synthesis. The Cu-MgO interaction on CuMgAl-x catalysts can be changed by changing the Mg/Al ratio. It was well known that catalysts with higher D_Cu_ are more dominant in stability, and smaller metal particles tend to provide more active sites, thus promoting adsorption and activation of reactants at the interface (Zhong et al., 2020). Figure 12 describes the mechanism comparison of CuMgAl-x catalysts for CO_2_ hydrogenation to methanol before and after the optimization of Mg/Al ratio. In this study, the diversity of basic sites on CuMgAl-x catalyst was affected by MSI. The interaction between Cu and MgO was enhanced when the Mg/Al ratio increased from 1 to 3. However, the number of basic sites of the catalyst decreased when further increasing the Mg/Al ratio, which due to the reduction of crystallinity of the hydrotalcite structure in the catalyst precursor by excess Mg. Therefore, the good alkalinity of the CuMgAl-3 catalyst was conducive to the adsorption of CO_2_. In addition, when the Mg/Al ratio was optimized to 3, the Cu with higher dispersion and smaller particle size was obtained on the CuMgAl-3 catalyst due to the strong interaction between Cu and MgO, which made H_2_ more easily dissociated, the overflow of H allows CO_3_* and HCO_3_^*^ to be quickly converted to HCOO^*^, which facilitates further conversion to CH_3_O^*^ and CH_3_OH. At the same time, the strong interaction between Cu and MgO was conducive to the formation of Cu^+^, which can inhibit the desorption of CO in RWGS reaction to a certain extent, and improve the selectivity of methanol. Therefore, the higher methanol production efficiency of CuMgAl-3 was the result of the adsorption of CO_2_ by more basic sites, the high H activation ability, and the inhibition effect of Cu^+^ on CO desorption.

## 4 Conclusion

In general, a series of CuMgAl-x catalysts with different Mg/Al molar ratios with hydrotalcite as the precursor were prepared, and the effect of Mg/Al molar ratios on the performance of CuMgAl-x catalysts for CO_2_ hydrogenation to methanol was studied. The Cu-MgO interaction on the CuMgAl-x catalyst can be regulated by changing the Mg/Al molar ratio, and the CuMgAl-3 catalyst showed the strongest Cu-MgO interaction. The strong interaction between Cu and MgO was conducive to increasing the number of basic sites and obtaining suitable acid sites. The strong Cu-MgO interaction was conducive to the formation of highly dispersed Cu, making H_2_ activation easier, accelerating the conversion of intermediate species CO_3_^2-^ and HCO_3_^*^to HCOO^*^, and facilitating further conversion to CH_3_O* and CH_3_OH. The strong interaction between Cu and MgO was conducive to the formation of Cu^+^, which can inhibit the desorption of CO in RWGS reaction, and improve the selectivity of methanol. Among all CuMgAl-x catalysts, CuMgAl-3 catalyst showed optimal performance for CO_2_ hydrogenation to methanol with the CO_2_ conversion rate (14.3%), methanol selectivity (94.5%), and STY of methanol(419.3 g⋅kg_cat._^−1^⋅h^−1^) at 240°C and 2.5 MPa.

## Data Availability

The original contributions presented in the study are included in the article/Supplementary Material, further inquiries can be directed to the corresponding author.
