# Supplementary material for: The influence of Mg/Al molar ratio on the performance of CuMgAl-x catalysts for CO2 hydrogenation to methanol
Source: Front Chem. 2024 Mar 14;12:1361930. doi: 10.3389/fchem.2024.1361930 (PMC10973157; doi:10.3389/fchem.2024.1361930)
Supplement: Supplementary file 1 [file DataSheet1.docx]

Supplementary Material

The influence of Mg/Al molar ratio on the performance of CuMgAl-x catalysts for CO_2_ hydrogenation to methanol

**Haoran Liu , Wenbin Huang, Zhen Xu, Yijing Jia, Meng Huang, Xiaoyue Liu, Han Yang, Rongrong Li, Qiang Wei, and Yasong Zhou^*^**

State Key Laboratory of Heavy Oil Processing, China University of Petroleum, Beijing, China

***Correspondence:** [zhouyasong2011@163.com](mailto:zhouyasong2011@163.com).


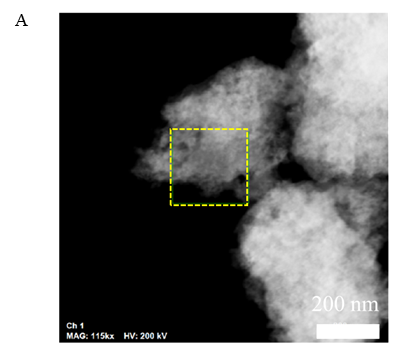

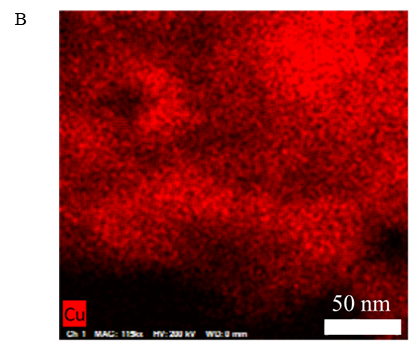

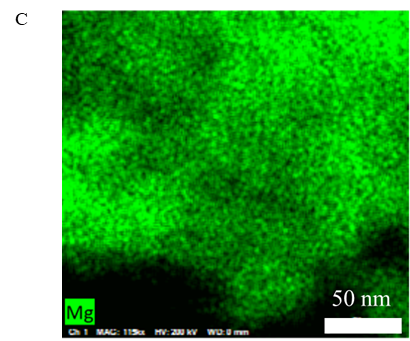

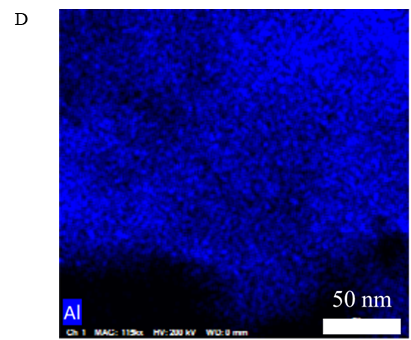

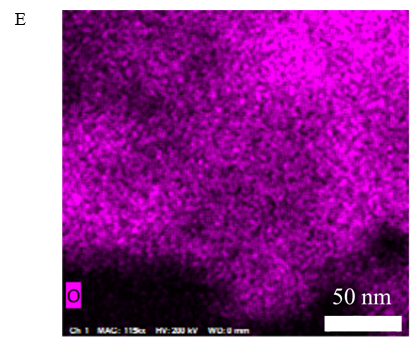


FIGURE S1. HAADF-STEM image (A) and corresponding elemental mappings images of Cu (B), Mg (C), Al (D), and O (E) of CuMgAl-3 catalyst

FIGURE S2. XPS spectra in the region of Cu LMM on the reduced CuMgAl-x catalysts

FIGURE S3. The catalytic performance of CuMgAl-3 catalyst over time

FIGURE S4. XRD of CuMgAl-3 catalyst (red-flesh, black-spent)


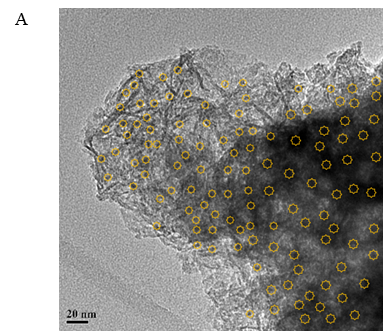


FIGURE S5. (A) TEM image and (B) Cu particle size distribution of spent-CuMgAl-3 catalyst

FIGURE S6. H_2_-TPD curves of the reduced CuMgAl-x catalysts

Table S1. Comparison of activity of CuMgAl-3 with other catalysts in CO_2_ hydrogenation to methanol

| **Catalysts** | **GHSV**  **(h^-1^)** | **P**  **(MPa)** | **T**  **(°C)** | **X_CO2_**  **(%)** | **S_CH3OH_**  **(%)** | **STY_CH3OH_**  **(g_MeOH_⋅kg_cat_^-1^⋅h^-1^)** | **Ref.** |
| --- | --- | --- | --- | --- | --- | --- | --- |
| Cu/ZnO/Al_2_O_3_ | 3600 | 3 | 240 | 16.2 | 63.8 | 130.0 | 1 |
| Cu/ZnO/ZrO_2_ | 3600 | 3 | 250 | 21.8 | 49.1 | 137.6 | 2 |
| Cu/ZnO/Al_2_O_3_/ZrO_2_ | 4000 | 5 | 190 | 10.7 | 81.8 | 87 | 3 |
| CuZnOZrO_2_MgO/Al_2_O_3_ | 1400 | 2 | 250 | 12.1 | 36.0 | 31.0 | 4 |
| CZZ@MgAlHT | 4000 | 3 | 250 | 4.5 | 83.4 | 64.5 | 5 |
| CuZnZr-MgAlLDH | 2000 | 3 | 250 | 4.9 | 78.3 | 36.5 | 6 |
| CuMgAlHT | 14963 | 2 | 260 | 4.0 | 50.8 | 192 | 7 |
| CU-0.5-300 | 21600 | 4.5 | 260 | 13.1 | 78.8 | 796.3 | 8 |
| Pd/In_2_O_3_ | 21000 | 5 | 300 | >20 | >70 | 889.6 | 9 |
| CuMgAl-3 | 9000 | 2.5 | 240 | 14.3 | 94.5 | 418.9 | This work |

1. Lei, H., Hou, Z., and Xie, J. (2016). Hydrogenation of CO_2_ to CH_3_OH over CuO/ZnO/Al_2_O_3_ catalysts prepared via a solvent-free routine. *Fuel* *164*, 191-198. doi: 10.1016/j.fuel.2015.09.082
2. Chen, H., Cui, H., Lv, Y., Liu, P., Hao, F., and Xiong, W., et al. (2022). CO_2_ hydrogenation to methanol over Cu/ZnO/ZrO_2_ catalysts: Effects of ZnO morphology and oxygen vacancy. *Fuel* *314*, 123035. doi: 10.1016/j.fuel.2021.123035
3. Xiao, S., Zhang, Y., Gao, P.; Zhong, L., Li, X., and Zhang, Z., et al. (2017). Highly efficient Cu-based catalysts via hydrotalcite-like precursors for CO_2_ hydrogenation to methanol. *Catal. Today* *281*, 327-336. doi: 10.1016/j.cattod.2016.02.004
4. Ren, H., Xu, C., Zhao, H., Wang, Y., Liu, J., and Liu, J. (2015). Methanol synthesis from CO_2_ hydrogenation over Cu/γ-Al_2_O_3_ catalysts modified by ZnO, ZrO_2_ and MgO. *J Ind Eng Chem* *28*, 261-267. doi: 10.1016/j.jiec.2015.03.001
5. Fang, X., Men, Y., Wu, F., Zhao, Q., Singh, R., and Xiao, P., et al. (2019). Moderate-pressure conversion of H_2_ and CO_2_ to methanol via adsorption enhanced hydrogenation. *Int. J. Hydrog. Energy* *44* (39), 21913-21925. doi: 10.1016/j.ijhydene.2019.06.176
6. Fang, X., Men, Y., Wu, F., Zhao, Q., Singh, R., and Xiao, P., et al. (2019). Improved methanol yield and selectivity from CO_2_ hydrogenation using a novel Cu-ZnO-ZrO_2_ catalyst supported on Mg-Al layered double hydroxide (LDH). *J CO_2_ Util.* *29*, 57-64. doi: 10.1016/j.jcou.2018.11.006
7. Cored, J., Mazarío, J., Cerdá-Moreno, C., Lustemberg, P. G., Ganduglia-Pirovano, M. V., and Domine, M. E., et al. (2022) Enhanced Methanol Production over Non-promoted Cu-MgO-Al_2_O_3_ Materials with Ex-solved 2 nm Cu Particles: Insights from an Operando Spectroscopic Study. *ACS Catal.* *12* (7), 3845-3857. doi: 10.1021/acscatal.1c06044
8. Liu, T., Hong, X., and Liu, G., (2020). In Situ Generation of the Cu@3D-ZrOx Framework Catalyst for Selective Methanol Synthesis from CO_2_/H_2_. *ACS Catal.* *10* (1), 93-102. doi: 10.1021/acscatal.9b03738
9. Rui, N., Wang, Z., Sun, K., Ye, J., Ge, Q., and Liu, C., (2017). CO_2_ hydrogenation to methanol over Pd/In_2_O_3_: effects of Pd and oxygen vacancy. *Appl. Catal. B: Environ.* *218*, 488-497. doi: 10.1016/j.apcatb.2017.06.069
